# P05.64. The role of self-discovery in women’s integration of CAM into their model of health care: qualitative data from a cohort study of 3,731 women

**DOI:** 10.1186/1472-6882-12-S1-P424

**Published:** 2012-06-12

**Authors:** V Murthy, D Sibbritt, J Adams, A Broom

**Affiliations:** 1 University of Newcastle, Newcastle, Australia; 2 Univeristy of Technology Sydney, Sydney, Australia; 3 University of Queensland, Brisbane, Australia

## Purpose

Research has established substantial levels of Complementary and Alternative Medicine (CAM) use alongside and concurrent to conventional health care use amongst mid-aged women. Yet, an examination of women’s CAM use in relation to notions of personal responsibility and self-discovery remains under-researched via large, nationally representative samples. The aim of this study is to examine the perceptions and experiences of mid-age women in relation to their CAM use.

## Methods

This study employs an inductive, thematic analysis of qualitative data obtained from an open text question completed by 3,731 women from a nationally representative sample (n=10,638) who participated in a survey as part of the Australian Longitudinal Study on Women’s Health. The focus of this study is the mid-aged group (between 56 and 61 years of age).

## Results

The analysis reveals that the women’s exploration of non-medical approaches to health and well being are driven by their commitment to personal inquiry, self-discovery and self-reflection. The women explain how their CAM exploration is fueled in part by their experiences of illness and those of others around them, which are interpreted as helping provide insights regarding empowerment and a holistic approach to health and healthcare.

## Conclusion

Australian women’s self-integrated models of health care emerging as a result of their health and illness experiences is compelling and cannot be ignored by General Practitioners, specialists and health policy makers. It is essential that the drivers of personal responsibility for health through non-medical approaches be subject to in-depth examination in order to aid and inform effective, responsive health policies around women’s CAM use.

